# Unveiling YKL-40, from Serum Marker to Target Therapy in Glioblastoma

**DOI:** 10.3389/fonc.2014.00090

**Published:** 2014-04-28

**Authors:** Fabio M. Iwamoto, Adília Hormigo

**Affiliations:** ^1^Department of Neurology and Brain Tumor Center, College of Physicians and Surgeons, Columbia University, New York, NY, USA; ^2^Department of Neurology, Icahn School of Medicine at Mount Sinai, New York, NY, USA

**Keywords:** YKL-40, glioblastoma, serum marker, prognosis, targeted therapy

## Abstract

Glioblastoma is the most common primary brain tumor in the adult and carries a poor prognosis with a median survival of only 14 months. Patients with glioblastoma are followed with MRI scans, but this technique has several limitations including low specificity to differentiate between tumor and treatment effect. Development of serum markers could significantly improve the care of glioblastoma patients. We review the current concept of developing YKL-40 as one of the most promising serum markers for glioblastoma, the recent advances on understanding the role of YKL-40 in gliomagenesis, and the promising evidence emerging from preclinical models on using this protein as a target for anti-glioma therapy.

## Introduction

Glioblastoma is the most common primary brain tumor in the adult and despite aggressive tumor resection and chemoradiation, the median survival of glioblastoma patients is only about 14 months (1). Although pathologically characterized by high tumor cell proliferation, necrosis, and neovascularization, glioblastomas are molecularly heterogeneous and highly dynamic tumors throughout the course of the disease. Patients with glioblastoma are followed clinically with MRI scans but this technique has several limitations including the inability to reliably distinguish between tumor progression and effects of treatment. Multiple brain tumor samplings during the disease course are also not feasible and less invasive tests such as serum markers could significantly improve glioblastoma patient care. YKL-40, a chitinase homolog also called human cartilage glycoprotein 39 or chitinase 3-like 1, is one the most promising serum markers for glioblastoma currently in development. YKL-40 is overexpressed in glioblastoma, secreted into the bloodstream, and easily and reliably measured in serum. Moreover, YKL-40 is involved in glioblastoma pathophysiology and may be a promising anti-glioblastoma therapeutic target.

## YKL-40 is a Glycoprotein Overexpressed in Glioblastoma

*CHI3L1/YKL-40* is one of the most differentially overexpressed genes in glioblastoma relatively to normal brain and low grade gliomas, as revealed by Tanwar et al. (2). There was no difference in expression between low-grade gliomas and normal brain (2). The overexpression of *YKL-40* mRNA in glioblastoma relatively to undetectable expression in low-grade gliomas and normal brain was confirmed by using Western Blot to measure the relative amounts of the YKL-40 protein. Furthermore, the YKL-40 glycoprotein could be detected in the serum of patients with glioblastoma and other high-grade gliomas (2–4). In children, however, YKL-40 is less often detected in glioblastoma suggesting that the underlying biology of glioblastoma in childhood might differ from the adult (5). Immunohistochemistry analysis detects YKL-40 expression mainly in the cytoplasm of tumor cells and reactive astrocytes, but the expression is low in macrophages and neurons mixed within the tumor (6, 7). The extracellular release of the protein into circulation suggests that the protein is a ligand, making it a potential target for neutralizing antibodies. Nevertheless, the receptors that YKL-40 might bind to initiate signaling transduction remain elusive, with the exception of endothelial cells, where YKL-40 is proposed to bind a membrane receptor syndecan-1 and integrin αvβ3 (8).

## Molecular Characteristics of Brain Tumors Expressing YKL-40

**Figure 1:**
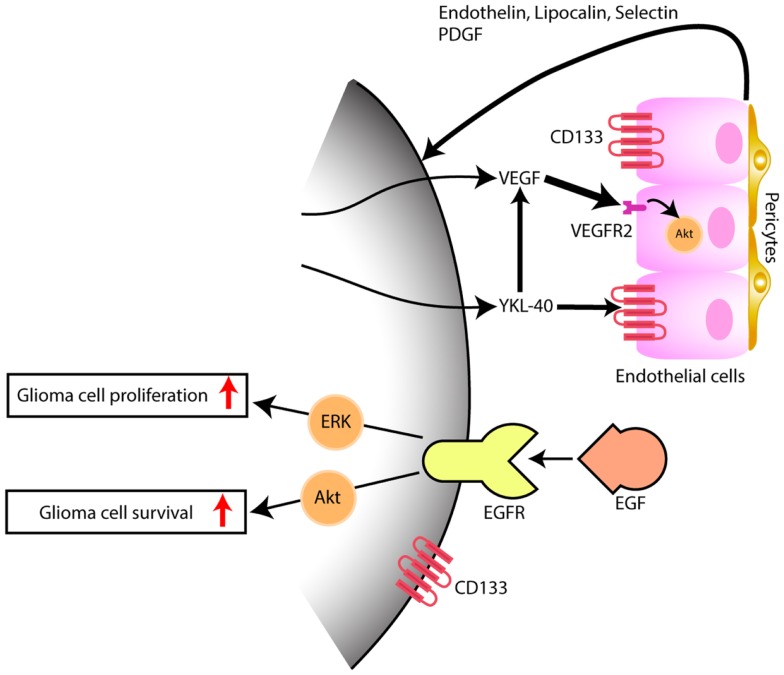
**Model depicting the interaction of tumor cells within the microenvironment, predominantly with endothelial cells**. While EGFR has been thoroughly and the most well studied and yet, not successful target signaling cascade in gliomas, YKL-40 may also promote glioma proliferation and survival, by inducing angiogenesis, through VEGF upregulation (thick arrow) and by VEGF-independent pathways (thin arrow), after persistent blockade of VEGF. Hypothetical, in a VEGF independent mechanism, YKL-40 secreted by the glioma cell modulates upon activation of CD133^+^ endothelial cells, the expression of endothelial-derived factors that are capable of triggering tumor angiogenesis and feedback to glioma cells, some of which also express the stem cell marker CD133^+^, promoting tumor growth.

The expression of YKL-40 in tumor xenografts obtained from the intracranial injection of cells dissociated from glioblastoma previously treated with chemotherapy or radiotherapy, and sorted for the stem cell marker CD133 was only detected in the CD133^+^ tumors (9). These CD133^+^ tumors had pathological characteristics consistent with glioblastoma with pseudopalisading necrosis and microvascular proliferation and stained for the endothelial cell marker CD31/PECAM-1 (9). In another report by Liu et al. that utilized the culture of glioblastoma dissociated cells under stem cell conditions with subsequent differentiation in the presence of serum, *EDN3* mRNA, known to be involved in the development of neural crest-derived cells lineages, and *CD133* mRNA were decreased under differentiation conditions while *EDN1* and *YKL-40* mRNA were upregulated (10). In this particular study, tumor xenografts generated by implantation of cells sorted according to CD133 expression, showed that *YKL-40* mRNA was found only on the tumors generated by the CD133^−^ cells. The discrepancy between the results of both works concerning the CD133 cell fraction expressing YKL-40 may be related to cell-lineage expression pattern of CD133 and to a different cell of origin for glioblastoma among the various glioblastoma subclasses, as we have previously pointed out (11). We and others have also found CD133 to be expressed in high grade glioma vasculature (10, 12). Additionally, we identified specific genes upregulated in CD133^+^ endothelium that code for signaling factors, such as endothelin, lipocalin, selectin, and PDGF that by themselves may be implicated on glioma angiogenesis, proliferation, and survival (10) (Figure 1).

YKL-40 expression was identified by the Cancer Genome Atlas to be a marker for the mesenchymal subtype of glioblastoma (13). The upregulation of YKL-40 characterizes primary glioblastoma and was not found in secondary glioblastoma, which showed a downregulation of the *CHI3L1* gene (14). *In vitro* studies showed that YKL-40 was associated with chromosome 10 loss, increased resistance to radiotherapy, capacity of invasion, and metalloproteinase activity (15, 16). In anaplastic oligodendroglioma, loss of heterozygosity in chromosome 10q correlated with increased YKL-40 expression but no correlation was found with 1p19q codeletion. YKL-40 expression was lower in tumors with *EGFR* amplification or EGFR immunostaining (7). Survival of patients who have a glioblastoma that does not express either YKL-40 or the EGFR variant, EGFRvIII, was longer and the disease had a better outcome (17).

## Detection of Circulating YKL-40 Protein in Patients with Glioblastoma

Circulating YKL-40, which is secreted by glioblastoma cells can be easily measured in serum through ELISA. There is no significant circadian variation in YKL-40 serum levels (18) and the protein levels are stable in collected blood for up to 7 days. A prospective longitudinal study correlated serum levels of YKL-40 with MRI findings in 197 patients with glioblastoma (4). Serum YKL-40 levels were significant lower in glioblastoma patients with no radiographic evidence of disease compared to those with radiographic evidence of tumor. Moreover, a doubling or higher increases of the serum YKL-40 level was seen in 47% of glioblastoma patients over time. Importantly, this increase in YKL-40 levels was independently associated with a shorter survival, and it was the most predictive prognostic marker when compared to age, extent of tumor resection or performance status (4).

A smaller study of 60 patients with glioblastoma who underwent gross total or subtotal resection showed that levels dropped postoperatively more often in patients who had more extensive tumor resections (19). Additionally, patients who had post-operatively an increase in YKL-40 serum levels by 100% or higher, had a very short median survival of only 76 days (19).

## YKL-40 Expression in Glioblastoma Tumor Tissue and Prognosis

Pelloski et al. reported on 140 glioblastoma patients who underwent gross-total resection and immunohistochemistry was performed on tumor tissue for YKL-40 expression (16). Eighty (57%) tumors were strongly stained (score 2+) for YKL-40, 37 (26%) had an intermediate level of staining (score 1+), and 23 (16%) were negative (score 0). Patients with YKL-40 scores of 0 in this group had a median overall survival of 116 weeks, compared to a median survival of 53 weeks for patients whose tumors had 1+ staining, and 41 weeks for those with scores of 2+.

Another independent study by Colman et al. using four datasets of mRNA expression in glioblastomas identified a 9-gene set, which included *YKL-40*, as the most predictive for prognosis among more than 10,000 genes evaluated by mRNA microarrays (20). This nine-gene set that included *YKL-40* was then validated looking at protein expression by immunohistochemistry on formalin-fixed glioblastoma tissue, which is more widely available compared to frozen tumor tissue required for mRNA expression (20).

Another study of 105 patients with glioblastoma showed that extent of resection, MGMT promoter methylation status and YKL-40 expression by immunohistochemistry were the most important prognostic factors in newly diagnosed glioblastomas (21).

## Regulation and Function of YKL-40 in Glioma

In recent years, advances have been made that shed light on the role of YKL-40 in glioma biology. Human embryonic kidney 293 cells transfected with *CHI3L1/YKL-40* became transformed and initiated tumors when transplanted into the rat brain (22). Stable transfection of astrocytes with *CHI3L1* conferred resistance to radiation and increased invasion capacity (15). In a chick chorioallantoic membrane assay, the inhibition of *VEGFA* by RNA interference in human glioma cells produced avascular tumors that revealed an upregulation of *CHI3L1/YKL-40* (23). Exposure of U87 cells to stress, including hypoxia, ionizing radiation, and chemotherapy with etoposide or serum depletion, resulted in increased YKL-40 levels in the culture media, suggesting that YKL-40 promoted survival of cancer cells in adverse conditions (24). Supporting these findings, the knockdown of *CHI3L1/YKL-40* reduced glioma invasion, increased cell death induced by cisplatin, etoposide, and doxorubicin, and decreased cell–matrix adhesion and expression of MMP-2 (25).

There is supporting evidence for the role of YKL-40 in glioma cell proliferation by activation of MAPK and AKT pathways (6). In fact, inhibition of *YKL-40* expression by siRNA, led to cell arrest in G1 and decreased activity of phospho-ERK1/2 and phospho-AKT (6). NF-κB pathway is also associated with *CHI3L1/YKL-40*. TNF-α recruits p65 and p50 subunits of NF-κB to the *CHI3L1/YKL-40* promoter, suppressing the expression of YKL-40 in glioma cell lines. TNF-α recruits histone deacetylases, HDAC1 and HDAC2, promoting deacetylation of histone H3 at the *CHI3L1/YKL-40* promoter in a cell type specific manner (26). REL B, a protein belonging to the NF-κB protein complex promotes expression of genes belonging to the mesenchymal glioblastoma subtype, including *CHI3L1/YKL-40* (27). Loss of REL B led to lower levels of YKL-40 protein and decreased tumor size, and when overexpressed, it upregulated *CHI3L1/YKL-40* mRNA (27). In contrast, TNF-α promotes mesenchymal transformation of a proneural glioma stem cell line and increase in YKL-40 and CD44 expression through activation of NF-κB. In addition, transformation from proneural to mesenchymal glioma stem cell line, increased radioresistance in an NF-κB dependent manner (28).

Overexpression of a splice variant of a transcription repressor NF-X, designated NFI-X3 (nuclear factor I-X3) enhanced YKL-40 expression in glioma cells by binding the regulatory elements of *CHI3L1/YKL-40* promoter, activating transcription, promoting invasion, and migration (29). Variant alleles of *CHI3L1/YKL-40* promoter GG, CC, and GC in tumors had no significant impact on the survival of patients, notwithstanding those that have GG variant tended to have longer survival (30).

## Strategies to Target YKL-40 in Glioblastoma

The first steps for therapy to target YKL-40 are on the way as the regulation and function of YKL-40 gets unveiled. Among possible approaches is modulation of NF-κB signaling and inhibition of ERK1/2 pathway. Resveratrol, a plant phenol inhibited, *in vitro*, the proliferation and invasion of U87 cells by decreasing the activity of *CHI3L1/YKL-40* promoter and lowering the levels of mRNA transcript and YKL-40 protein expression (31). Floyd et al. (32) found that YKL-40 may be involved in the Notch pathway by inhibiting cleavage of Notch1 by treating cells with a α-secretase inhibitor, INCB3619. The authors also showed that there was an increase in survival and decrease in tumor size of their xenograft tumor model when treated with the α-secretase inhibitor, by using nanoparticles as the drug delivery system (32). They saw no effect when a γ-secretase inhibitor was used instead. Okada et al. (33) conducted a phase I/II trial to evaluate the safety and immunogenicity of a vaccine using polarized dendritic cells loaded with polyinosinic–polycytidylic acid stabilized by lysine and carboxymethylcellulose (poly-ICLC) and synthetic peptides for glioma-associated antigens including YKL-40. The vaccine was well tolerated, immunogenicity was developed, and preliminary clinical activity was seen.

More recently, it has been shown that YKL-40 is associated with tumor angiogenesis. This is particularly interesting in glioblastoma, which is one most vascularized human cancers, and vascular proliferation is one of its pathological hallmarks. Francescone et al. (34) showed that YKL-40 upregulated vascular endothelial growth factor (VEGF), which is considered the primary promoter of angiogenesis in glioblastoma. More specifically, YKL-40 was found to induce interaction of the membrane receptors syndecan-1 and integrin αvβ5, and triggered a signaling cascade through FAK to ERK-1 and ERK-2, leading to elevated VEGF expression and enhanced angiogenesis (34). Both VEGF and YKL-40 had synergistic effects on tumor angiogenesis, however persistent blockage of VEGF led to upregulation of YKL-40, supporting that glioblastoma eventually develop VEGF-independent pathways of tumor vascularization (Figure 1); this may be one of the mechanisms that explains why glioblastoma always becomes resistant to anti-VEGF treatment such as bevacizumab. Faibish et al. (35) developed a YKL-40 monoclonal neutralizing antibody, mAY that blocked, in a dose dependent manner, tube formation induced by U87 conditioned medium expressing YKL-40. Tumors obtained in xenografts by subcutaneous implantation of U87 cells into SCID/Beige mice that were subsequently treated with mAY antibody, had smaller volume and exhibited decreased angiogenesis. Moreover, preclinical studies suggest (36).

In conclusion, glioblastoma is a heterogeneous tumor with multiple pathways that have a different relevance and implication in tumorigenesis among tumors with the same histological pattern. YKL-40 as a serum marker possibly reflects the biology not only of tumor cells but also of the microenvironment (Figure 1). Endothelial cells in the tumor microenvironment, likely, may have various levels of heterogeneity among the glioblastoma tumors. Angiogenesis therapeutic is complex, and future trials may need to combine anti-VEGF treatment with anti-YKL-40 target therapy to control glioma cell growth with inactivation of angiogenic factors. In addition, YKL-40 is involved in the pathogenesis of the mesenchymal glioblastomas, which are the most aggressive molecular subtype of these tumors. Further studies are needed to evaluate if longitudinal serum YKL-40 measurements could be a reliable marker for diagnosis of glioma transformation from the less aggressive proneural subtype to the more aggressive mesenchymal subtype.

## Conflict of Interest Statement

The authors declare that the research was conducted in the absence of any commercial or financial relationships that could be construed as a potential conflict of interest.
